# Dynamic protein ligand interactions – insights from MS

**DOI:** 10.1111/febs.12707

**Published:** 2014-01-21

**Authors:** Carla Schmidt, Carol V. Robinson

**Affiliations:** ^1^ Department of ChemistryUniversity of OxfordUK

**Keywords:** crosslinking, hydrogen–deuterium exchange, hydroxyl radical footprinting, MS, protein complexes, protein–ligand interactions, proteomics

## Abstract

Proteins undergo dynamic interactions with carbohydrates, lipids and nucleotides to form catalytic cores, fine‐tuned for different cellular actions. The study of dynamic interactions between proteins and their cognate ligands is therefore fundamental to the understanding of biological systems. During the last two decades MS, and its associated techniques, has become accepted as a method for the study of protein–ligand interactions, not only for covalent complexes, where the use of MS is well established, but also, and significantly for protein–ligand interactions, for noncovalent assemblies. In this review, we employ a broad definition of a ligand to encompass protein subunits, drug molecules, oligonucleotides, carbohydrates, and lipids. Under the appropriate conditions, MS can reveal the composition, heterogeneity and dynamics of these protein–ligand interactions, and in some cases their structural arrangements and binding affinities. Herein, we highlight MS approaches for studying protein–ligand complexes, including those containing integral membrane subunits, and showcase examples from recent literature. Specifically, we tabulate the myriad of methodologies, including hydrogen exchange, proteomics, hydroxyl radical footprinting, intact complexes, and crosslinking, which, when combined with MS, provide insights into conformational changes and subtle modifications in response to ligand‐binding interactions.

**Abbreviations**
ABC: ATP‐binding cassette
AP: affinity purification
ATPase: ATP synthase
cATPase: chloroplast ATP synthase
CCS: collision cross‐section
CID: collision‐induced dissociation
DSPC: 1,2‐distearoyl‐sn‐glycero‐3‐phosphocholine
EM: electron microscopy
GPCR: G‐protein‐coupled receptor
HX‐MS: hydrogen exchange monitored with MS
IM: ion mobility
*K*_D_: dissociation constant
LC‐MS/MS: liquid chromatography‐coupled MS
OmpF: outer membrane porin F
P‐gp: P‐glycoprotein
POPA: 1‐palmitoyl‐2‐oleoyl‐sn‐glycero‐3‐phosphate
TTX: tentoxin
U‐snRNP: uridine‐rich small nuclear ribonucleoprotein particle

## Introduction

MS is well established as an analytical technique with a mass range encompassing single atoms through to complex polymers of several million daltons. Within this mass range from tens to several million daltons, many interesting biological assemblies reside. During the last two decades, MS has become indispensible for the study of proteins and their assemblies. Many of these developments emanated from the discovery of two ‘soft’ ionisation techniques (ESI 1 and MALDI 2). Whereas established ionisation techniques in the early 1990s caused fragmentation of peptides and proteins, the invention of these two techniques made possible the study of proteins and peptides without derivatisation, thus leading to developments in proteomics. In its simplest inception, proteomics is used to identify proteins by sequencing peptides derived by enzymatic cleavage. Nowadays, however, proteomics also involves the determination of post‐translational modifications and quantification of proteins, making it invaluable for the description of cell signalling pathways and biomarker discovery.

In line with developments in proteomics, MS is also gaining in importance in structural biology (see 4 for recent reviews). A multitude of techniques have evolved to address different questions in the structural elucidation of protein–ligand assemblies. In most cases, these techniques involve studying proteins after modification (e.g. labelling of accessible amino acids) and subsequent hydrolysis to form peptides that are then studied under denaturing conditions. However, an alternative and unique way to study proteins is from their native state, introduced from aqueous buffered solutions. Nondenaturing or native MS, as it is sometimes known, enables the study of intact protein–ligand complexes in the gas phase of a mass spectrometer. Although early studies using this technique focused on relatively low molecular mass single proteins and ligands with a range of affinities 7, there have been great improvements in instrumentation over the years, such that the study of complexes in the megadalton range is now possible 9.

Although the mass range of the complex is rarely an issue in getting soluble proteins into the gas phase, resolution in the resulting mass spectrum is a key criterion when protein–ligand interactions are studied. For example, it is a simpler task to resolve a relatively small ligand (~ 300 Da) when it is in complex with a small protein of < 10 kDa than to resolve a similar‐sized ligand within a megadalton ribosome complex. Moreover, recent discoveries of new ways of transporting membrane proteins into the gas phase from detergent micelles bring their own challenges. Resolving small molecules within the context of the vast excess of detergent molecules that surround the membrane complex and subsequently distinguishing detergent molecules from small‐molecule ligands is particularly challenging.

Here, we give an overview of MS techniques that are commonly applied to study protein–ligand interactions. Specifically, we include proteomics studies, hydrogen–deuterium exchange, hydroxyl radical footprinting, chemical crosslinking, and MS of intact complexes (Table 1). We discuss some of the technical difficulties that have to be overcome, and highlight the advantages and pitfalls of the various approaches. Although this is by no means an exhaustive list, we also highlight many of the exciting insights that have been made possible by application of these various techniques.

**Table 1:** An overview of methods used in structural MS to study protein–ligand complexes. The principles of the methods and the expected outcomes are described. Examples are given for each method.

Method	Principle	Outcome	Examples
Proteomics	Digestion, LC‐MS/MS analysis of generated peptides, and database search of proteins from (purified) protein complexes	Identification of proteins in protein assemblies	Spliceosomal complexes 87
Quantitative proteomics	Labelling of proteins/peptides or label‐free approaches to compare or absolutely quantify proteins	Comparison of different complex assembly states. Identification of specific and nonspecific binders (relative quantification). Protein stoichiometries (absolute quantification)	Spliceosomal complexes 88, chromatin binders 18, hPrp19–CDC5L complex 16, Ser/Thr protein phosphatase 5 interactome 19
Hydrogen–deuterium exchange	Solvent‐accessible backbone hydrogens exchange with deuterium atoms from ‘heavy’ water. Analysis of intact proteins reveals differences (e.g. folded/unfolded state), and digestion and LC‐MS/MS analysis uncover protein sites that undergo exchange	Solvent accessibility	Calmodulin–Ca^2+^ interactions 27, viral capsids 28, SH3 domains 90
Hydroxyl radical footprinting	Hydroxyl radicals react with accessible amino acid side chains to form oxidised residues. After digestion, the modified peptides (residues) are identified by LC‐MS/MS	Solvent accessibility	Cytochrome *c* folding 33, serotonin receptor 37
Crosslinking			
Chemical crosslinking	Bifunctional crosslinkers covalently link functional groups of neighbouring proteins. After digestion, crosslinked residues are identified by LC‐MS/MS and database search	Protein–protein interaction sites, distance restraints	Phosphatase 2A protein network 45, RNA polymerase II–TFIIF complex 46
UV crosslinking	RNA (DNA) bases are excited by UV irradiation to form covalent bonds between bases and proteins in close proximity. Proteins and RNA are digested, and LC‐MS/MS analysis of the protein–RNA conjugate reveals the peptide sequence and the crosslinked RNA (DNA) base	Protein–RNA/DNA interaction sites	NusB–S10 91, ASH1–mRNA 92
Native MS	MS analysis of intact protein complexes by the use of mass spectrometers modified for transmission of large protein assemblies	Protein stoichiometries, topology, heterogeneity, protein interactions, ligand interactions, stable protein subcomplexes	Ribosomes 10, viruses 56, ATPases 47
IM‐MS	Determination of the drift time of proteins and protein complexes in the IM cell of the mass spectrometer, and conversion into CCSs	Shape/conformation of proteins and protein complexes. Conformational changes	TRAP complex 61

## Identifying interactions through proteomics

Proteomics at its inception was defined as the study of the proteome of a cell or an organism under a set of controlled conditions. Today, proteomics not only involves relatively straightforward protein identification, but also, increasingly, simultaneous quantification and identification of post‐translational modifications. Moreover, and pertinent to this review, proteomics has also been used to study protein complexes in terms of their composition, subunit stoichiometry, and interactions (reviewed in 12). These studies involve complexes composed of just a few protein subunits up to large protein assemblies obtained after affinity purification (AP). The initial focus of these investigations was the identification of the subunit composition. However, the establishment of quantitative MS has greatly increased the application to protein complexes, as it allows comparison of different assemblies (reviewed in 13).

Consequently, in recent studies, labelling‐based and label‐free absolute and relative quantification have been performed to compare assembly states and to determine the subunit stoichiometries in purified protein complexes. An example of this approach was its application to different assembly intermediates of the spliceosome during its catalytic cycle. Protein subunits were quantified and compared with electron microscopy (EM) images to define the composition of particles by semiquantitative peptide/spectral counting (e.g. 15). Of particular interest was the characterisation of the human spliceosomal hPrp19–CDC5L complex, which consists of seven individual proteins and plays a crucial role in the assembly of the catalytically active spliceosome during pre‐mRNA splicing. By the use of synthetic peptides to match sequences derived from the different subunits, absolute intensities of the various subunits were defined, enabling stoichiometries to be derived 16. The hPrp19–CDC5L complex has also been used in a recent study to prove label‐free quantification techniques that are suitable for protein complex determination 17.

Distinguishing specific from nonspecific binding proteins has long been problematic when large protein interaction networks are defined. Although interactions can be identified readily, following AP coupled with MS, relative quantification is needed to distinguish between specific and nonspecific binders. Furthermore, quantitative AP coupled with MS allows the monitoring of dynamic and transient interactions in large protein assemblies. This was used to good effect in studies of chromatin, wherein specific protein binders were defined and assembled into complexes 18, and a novel phosphatase interaction partner, which acts as an Hsp90 cochaperone, was identified as a specific binder despite the fact that it is usually considered to be a background contaminant 19.

Accordingly, MS‐based proteomics is capable of identifying interaction partners in protein assemblies, and, in conjunction with MS of intact complexes, of confirming subunit stoichiometries or of unambiguously identifying specific interaction partners.

## Probing dynamic protein–ligand interactions with HX‐MS

HX‐MS was first employed back in the early 1990s to probe solution structure 20, to follow protein folding reactions 21, and to investigate enhanced stability as a function of ligand binding 7. Significant improvements in technology over the years have led to the technique becoming more robust, and to the development of hardware to facilitate experiments 22. The underlying methodology exploits the fact that amide protons of the protein backbone exchange with protons in aqueous solutions. The use of ‘heavy’ water (D_2_O) leads to the exchange of solvent‐accessible amide protons for deuterium. Although many different factors affect hydrogen exchange, including temperature, pH, solvent accessibility, and hydrogen bonding (reviewed in 23) the first two of these can be easily controlled. Solvent accessibility and hydrogen bonding are structure‐specific, and are thus indicators of conformation or conformational changes in proteins.

Hydrogen exchange is usually initiated by the dilution of folded proteins with D_2_O‐containing buffers, and the reaction is quenched by decreasing the pH to > 2.5. The labelling reaction is monitored at different time points, and the level of deuterium incorporation gives information about the accessibility or structural flexibility of the protein–ligand complex. As the deuterated and nondeuterated proteins differ in mass but have the same ionisation properties, MS is an ideal method to assess the extent of labelling. Global analysis of the undigested protein allows comparison of labelled and nonlabelled proteins in their folded and unfolded states. Digestion of the proteins post‐labelling and subsequent analysis of the peptides generated provides information on the particular sites of the protein that have undergone hydrogen–deuterium exchange. Note that the quenching conditions require the use of hydrolases that are active at low pH, and the use of pepsin is therefore favoured in HDX‐MS experiments 20. Fragmentation methods that prevent proton ‘scrambling’ (i.e. proton migration during backbone cleavage), such as electron capture dissociation and electron transfer dissociation, are preferred over collision‐induced dissociation (CID), and can allow residue‐specific resolution 24.

**Figure 1:**
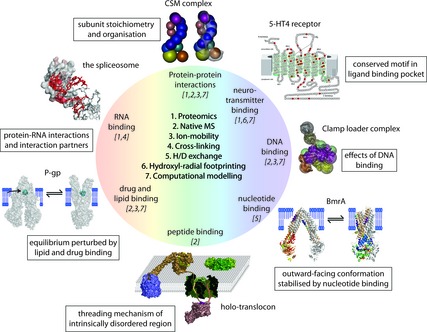
Structural MS and its various techniques. A multitude of structural MS techniques 1 have been applied and combined to study protein–ligand complexes – most of these have been coupled with computational modelling 7. Some recent examples are shown. The subunit stoichiometry and organisation of the CSM complex have been obtained from proteomics and IM‐MS of the intact complex. A model compatible with the available EM density map could thus be obtained [81]. Hydroxyl radical footprinting gave insights into neurotransmitter binding to the GPCR 5‐HT4. The schematic shows the GPCR with amino acids that were studied highlighted in red 33. The clamp loader complex was studied with IM‐MS and computational modelling, allowing construction of a three‐dimensional model of the fully assembled complex 59. Hydrogen–deuterium exchange was applied to study the effect of nucleotide binding on the conformation of the BmrA transporter. Hotspots for hydrogen‐deuterium exchange are represented by colour coding from cold to hot (blue < green < yellow < red) 25. Peptide binding probed by MS uncovered the threading mechanism through the bacterial OmpF 71. IM‐MS gave insights into specific lipid and drug binding to the P‐gp transporter 61. RNA–protein interactions in various spliceosomal complexes have been determined by combining UV‐crosslinking and proteomics. The model derived for the U1‐snRNA (red) in complex with U1‐specific proteins (grey space‐filling) is shown.

HDX‐MS has successfully been applied in several studies to analyse the structure of protein–ligand complexes. With this method, insights have been gained for calcium binding to calmodulin 27, protein–protein interactions during maturation of the HIV capsid 28, and open and closed conformations of an ATP‐binding cassette (ABC) transporter (BmrA) in the presence of a nucleotide ligand 29 (Fig. 1).

## Hydroxyl radical footprinting

Similar to hydrogen–deuterium exchange, hydroxyl radical footprinting when coupled with MS reveals the accessibility of proteins during folding and in dynamic interactions with ligands. Surface‐accessible amino acid side chains react with hydroxyl radicals to form the respective oxidised analogues, which can be identified after digestion of the proteins and analysis of the peptides generated. Hydroxyl radical footprinting thus generates nonspecific labels, and provides structural information at the single amino acid level (for reviews, see 30).

Hydroxyl radicals can be generated by transition metal‐dependent chemistry from peroxide (Fenton chemistry), photolysis of peroxide, or radiolysis of water. Fenton chemistry is a simple and inexpensive technique, but does require longer reaction times than photolysis or radiolysis approaches. In contrast, laser‐induced dissociation of hydrogen peroxide and high‐flux X‐rays from synchrotron sources generate hydroxyl radicals on microsecond to millisecond timescales, allowing for kinetic studies (e.g. 32). The hydroxyl radicals react with solvent‐accessible amino acid side chains of the proteins in solution. The relative reactivity of the amino acid side chains has been established, with cysteine being the most reactive and glycine being the least 34. The mechanism of the modification depends in part on the side chain chemistry of the amino acids, but mainly relies on incorporation of hydroxyl or oxo groups, generating a mass shift of +16 or +14 atomic mass units respectively.

Hydroxyl radical footprinting experiments typically include first the exposure of the protein complex, and then digestion into peptides and analysis by MS. Unmodified and modified peptides are usually quantified according to extracted ion current values, and the rate of modification can be calculated from the dose–response curve plotted for each peptide as a function of exposure time 30. This technique has been successfully applied in many studies, e.g. to map the G‐actin‐binding surface of cofilin 35 or Ca^2^^+^ ‐dependent structural changes of gelsolin 36. From the use of hydroxyl radical footprinting together with computational modelling, a structure of an antagonist‐bound G‐protein‐coupled receptor (GPCR) was also proposed (Fig. 1) 37.

## Probing changes in interface interactions with chemical crosslinking

Chemical crosslinking is a straightforward approach for studying the three‐dimensional structure of proteins and protein complexes. Functional groups of neighbouring proteins are covalently linked by the use of bifunctional crosslinking reagents, and MS of the crosslinked peptides, after digestion of the proteins, identifies the crosslinked peptides. The length of the crosslinker defines a distance constraint between crosslinked amino acids that allows deductions to be made concerning the three‐dimensional arrangement of proteins in noncovalent assemblies (reviewed in 38).

A broad range of crosslinking reagents, specific for different functional groups of amino acid side chains, are available commercially and numerous protocols have been established (reviewed in 40). However, the analysis of crosslinked proteins/peptides remains challenging; a simple database search cannot be applied, as crosslinked peptides can be derived from different subunits and different regions of proteins, such that peptides cannot be aligned with the sequence as such. The use of stable isotope‐labelled crosslinking reagents in a 1 : 1 ratio facilitates the identification of crosslinked species, as a pair of peaks in the spectrum facilitates detection and validation during data analysis 41. Recent developments in instrumentation and data analysis tools (e.g. 42) have further increased the potential of chemical crosslinking and MS to be used for the study of large protein assemblies and networks (e.g. the phosphatase 2A protein network 45 or the RNA polymerase II–TFIIF complex 46). The use of differentially labelled crosslinkers to compare different functional states of protein complexes has recently been introduced to probe the effects of post‐translational modifications on nucleotide binding in an intact F_1_F_0_‐ATPase 47. The technical limitations of such an approach were also investigated with human serum albumin as a model system 48. The ability to quantify changes in interactions in response to different stimuli provides new possibilities for studying conformational changes in response to ligand‐binding interactions.

In addition to chemical crosslinking, UV‐induced crosslinking (also termed photo‐crosslinking) can be applied to study protein–protein interactions. The underlying principles involve insertion of photoreactive groups into proteins by absorption of UV light, without causing damage to the protein itself, while generating the reactive crosslinking group. Most of these photolabile precursors are azides, diazirines, diazo compounds, and benzophenones. Upon UV irradiation, these precursors react nonspecifically with CH and NH groups of the protein backbone to form covalent, stable bonds that are suitable for detection by MS (for a review, see 39). In this way, ligand‐dependent conformational changes of peroxisome proliferator‐activated receptor were studied using *p*‐benzoylphenylalanine, which was genetically encoded into the ligand‐binding pocket 49. Photoreactive amino acids harbouring a diazirine moiety (photo‐leucine, photo‐isoleucine and photo‐methionine) can also be applied in a similar manner 50. The great advantage of these amino acids is that they are not recognised by the cell's translation machinery, allowing for metabolic labelling of the proteins and thus enabling *in vivo* crosslinking. Furthermore, as these hydrophobic amino acids are often present in transmembrane domains, these crosslinkers broaden the scope of crosslinking from the traditional soluble proteins to encompass those that are membrane‐embedded.

Whereas chemical crosslinking is primarily employed to study protein–protein interactions, UV‐induced crosslinking can be applied to study protein–RNA and protein–DNA interactions. Upon UV irradiation, the nucleobase is excited, and can form covalent bonds with amino acids in close spatial proximity. Subsequent hydrolysis of the protein and nucleic acid moiety leads to peptide–RNA/DNA conjugates, which are then purified and analysed with MS to identify the crosslinked residues (see 51 for a recent review). Protein–RNA interactions within spliceosomal complexes and subcomplexes [uridine‐rich small nuclear ribonucleoprotein particles (U‐snRNPs)], including U1 and U4/U6.U5 snRNPs, were studied in this way 52, revealing binding sites of the Sm proteins on the U‐snRNA 53. Enrichment strategies facilitated the identification of protein–RNA crosslinks by MS, and allowed sequence information to be obtained for both the peptide and the RNA moieties 54. The use of photoreactive base analogues, together with the development of software for automated crosslinking analysis, has further improved the protein–RNA crosslinking strategy 55. Collectively, these developments highlight the potential for studying large protein–RNA assemblies with photo‐crosslinking and MS.

## Shapes and sizes from ion mobility (IM)‐MS of intact protein complexes

Nondenaturing or native MS of intact protein–ligand complexes can reveal their composition, heterogeneity, stoichiometry, topology, and subunit interactions (reviewed in 9). Microlitre quantities of protein complexes are required at micromolar concentrations. The mass range can encompass small ligands through to large protein assemblies, often within the same mass spectrum. One of the prerequisites of the method is keeping the proteins in their native state and avoiding unfolding of subunits during transfer into the gas phase. This is achieved primarily by using volatile aqueous buffers such as ammonium acetate, rather than the organic solvents typically used in conventional MS. Impressive examples include intact ribosomes 10, viruses 56, and ATPases 57.

Modifications of the mass spectrometer are often necessary to obtain optimal conditions for the survival and transmission of protein complexes 58, with new instrumentation modified for this purpose coming to the fore 59. One caveat regarding the study of protein–ligand complexes with this approach concerns the concentration range suitable for analysis by MS. If the *K*_D_ value for a given protein ligand is above the micromolar concentration typically used for electrospray, then the protein will be ligated in solution prior to electrospray. If, however, the complex has a low millimolar *K*_D_ value, the extent of complex formation in solution will be low, and the population of ligated protein may be difficult to detect. The range of acceptable *K*_D_ values was exemplified recently in a study of Hsp90 and three of its cochaperones, wherein *K*_D_ values ranging from low micromolar to high nanomolar were derived 60.

MS of intact complexes can be complemented by IM and computational modelling approaches to provide topological information. Drift times recorded in IM experiments can be converted into collision cross‐sections (CCSs), which, in turn, can be related to the conformation of the macromolecular assembly. One of the earliest IM‐MS studies showed that the native structure of a ring‐shaped protein complex could be preserved in the gas phase of the mass spectrometer, and that the CCS measured with IM‐MS was in accord with that calculated from the crystal structure 61. Nowadays, it is possible to use IM CCS as a restraint to model protein subcomplexes into EM density maps of large protein assemblies 62.

## Integrating MS data by the use of computational modelling

The gold standard for determining high‐resolution structures of proteins and their complexes is undoubtedly X‐ray crystallography and, for small proteins that are soluble at high concentrations, NMR spectroscopy. However, these techniques are dependent on relatively high purity and abundance, which is not always possible for protein assemblies isolated directly from cells without overexpression or from tissue or organisms. Integration of data from a number of methods is often required to obtain structural models of protein–ligand complexes from these sources. Integrative modelling approaches are therefore required to optimise the modelling process (reviewed in 64).

MS of intact complexes and subcomplexes can provide subunit connectivity maps, whereas crosslinking yields spatial restraints and parameters for docking. Different strategies are then followed, involving either template‐based modelling using structures of homologous complexes (e.g. 65), or protein docking relying on possible assembly configurations of the components 66. Impressive examples include the nuclear pore complex 67, the 26S proteasome holocomplex 68, and the clamp loader complex 69 (Fig. 1).

Having established the methodology that underpins applications of MS, we now focus on protein–ligand complexes classified according to the ligand, and highlight the value of the various approaches described.

## Quantifying lipid binding to membrane protein complexes

**Figure 2:**
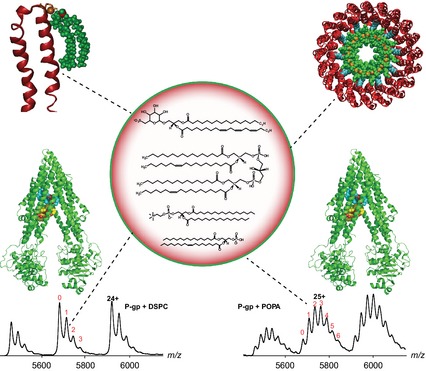
Lipid binding to membrane protein complexes studied by MS. A sulfolipid was shown to bind specifically in the inner membrane ring of cATPase. The upper left model shows how the lipid is attached to the membrane ring subunit. Cardiolipins were found to bind to the membrane ring of V‐type ATPase in *E. hirae*. The ‘lipid plug’ reduces the inner cavity of the membrane ring to stabilise binding of the central stalk (upper right panel). MS reveals that the ABC transporter P‐gp binds up to six negatively charged diacylglycerides (e.g. POPA; lower right panel), which bind more favourably than zwitterionic lipids (e.g. DSPC; lower left panel).

Ever since the earliest mass spectra of membrane complexes were recorded, it has been apparent that lipid binding is preserved in the gas phase 70. To exploit the opportunities that this presents, we investigated lipid binding to the ABC transporter P‐glycoprotein (P‐gp), to establish a range of lipid‐binding affinities and to determine *K*_D_ values 71. Following a gas‐phase activation strategy, developed in our laboratory to remove detergent micelles from membrane protein complexes in the gas phase 72, we acquired mass spectra of P‐gp. An unusual attachment of detergent molecules was revealed after release from the micelle, consistent with the ability of this pump to incorporate detergent molecules in its ligand‐binding cavity. Incubation with phosphoglyceride lipids, however, revealed that lipid molecules bind more favourably to the intact protein complex than detergent molecules (Fig. 2). Measurement of the rates of lipid binding and calculation of apparent *K*_D_ values showed that lipid binding is specific, and that up to six negatively charged diacylglycerides bind more favourably than zwitterionic lipids (Fig. 2). Similar experiments with cardiolipins confirmed their binding, and showed that this small‐molecule binding can perturb the equilibrium between inward‐facing and outward‐facing states, as demonstrated by IM‐MS 71.

Turning to much larger assemblies, specific lipid binding was also observed in the membrane ring of V‐type and F‐type ATP synthases (ATPases) 47. After tryptic digestion of the proteins, the protein–lipid mixture was subjected to liquid chromatography‐coupled MS (LC‐MS/MS), and singly charged lipids were identified manually from MS and MS/MS spectra. Interestingly, in both cases (V‐type and F‐type ATPases), lipids were identified that were not abundant in their respective membranes but were specific for the ATPases. Predominant lipid molecules were: cardiolipins and phosphatidylethanolamine (V‐type ATPases, *Enterococcus hirae* and *Thermus thermophilus*, respectively) and sulfoquinovosyl diacylglycerol (F‐type ATPase, spinach chloroplasts). By detection of the membrane ring with bound lipids as an intact complex and the use of standard lipids, the protein/lipid stoichiometry was determined in different species. This information was then used in a docking approach to place lipids within the membrane ring (Fig. 2). The cavities of all rings were reduced dramatically by the ‘lipid plug’, which stabilised binding of the central stalk within the ring of the ATPases 47.

New opportunities are provided by recent discoveries of lipid binding to protein complexes within nanodiscs and micelles. Nanodiscs have been studied by the use of hydrogen–deuterium exchange and CID, which revealed the conformation of the membrane structural proteins and the cohort of lipids within nanodiscs, respectively 73. When intact protein complexes are ejected from these lipidic environments, protein–lipid interactions are preserved, implying that nanodiscs will provide an excellent opportunity for probing drug binding to membrane protein complexes 75.

## Nucleic acid or nucleotide binding – distinguishing specific from nonspecific binding

**Figure 3:**
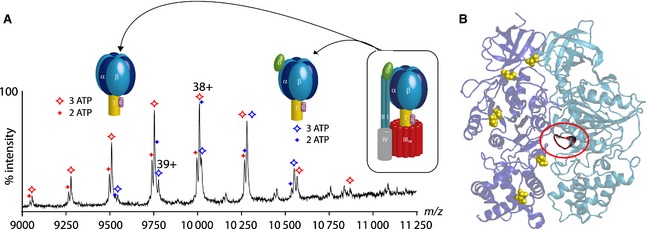
Nucleotide binding and occupancy of cATPase. (A) Mass spectrum of the intact F_1_‐ATPase and F_1_–δ‐ATPase. Peaks show splitting with a mass difference that can be attributed to the loss of ATP/ADP. Assignment of the peaks reveals populations of ATPases with two and three bound nucleotides. (B) The structure of the β/α‐interface. The catalytic binding site (P‐loop) is highlighted in red. A series of phosphosites were identified in the α‐subunit and β‐subunit (shown as yellow space‐filling), prompting the proposal that phosphorylation status controls access to nucleotide‐binding sites.

Nucleotide binding can be studied similarly to lipid binding, but the results have to be interpreted with care, owing to the ability of nucleotides to undergo nonspecific addition to basic proteins. Careful data analysis has to be applied to distinguish specific from nonspecific binding 76. A mass spectrum of the chloroplast F_1_‐ATPase, isolated directly from spinach, revealed subcomplexes of the F_1_ moiety containing two and three bound ATP molecules. Using a previously published assignment strategy 77, we determined the maximum number of bound nucleotides to be three (Fig. 3A). The location of phosphorylation sites equidistant along one face of the α/β‐interface suggested their role in controlling this interface (Fig. 3B). Dephosphorylation with a phosphatase led to depletion of nucleotides, with populations of molecules containing one, two and three nucleotides. The location of phosphosites and their effect on nucleotide occupancy prompted the proposal that interactions within the α/β‐interfaces are weakened after dephosphorylation, leading to loss of nucleotides 47.

Quantitative proteomics has recently been applied to distinguish specific protein binding from background binding to RNA motifs. For this purpose, bait and control RNA sequences, which either contained or did not contain the specific RNA motif, were incubated with light (unlabelled) and heavy (labelled with stable isotopes) cell populations, by use of a technique known as stable isotope labelling by amino acids in cell culture. Background protein binding occurred to an equal extent in both samples, whereas proteins that bind specifically showed enrichment in the pulldown experiments with the bait RNA 78.

## Peptide binding – obtaining mechanistic insights

Peptide binding to proteins or their complexes is of particular importance, for several reasons. First, peptides represent an important class of neurotransmitters, and thus regulate specific functions in the nervous system; and second, synthetic peptides can be used to study protein–protein interaction interfaces in detail. The latter have been used frequently to probe specific binding (binding motifs), structural dynamics, or cellular mechanisms.

Bringing together photoaffinity labelling and chemical crosslinking led to an elegant study of Ca^2^^+^‐dependent Munc13–calmodulin interactions 79. Photoreactive Mun13 peptides were generated by replacing potential anchor residues, and covalent Munc13(peptide)–calmodulin complexes were obtained in the presence of Ca^2+^ after irradiation with UV light. Covalent complexes were then characterised with SDS/PAGE and MS to identify Munc13‐binding sites. This strategy was further combined with chemical crosslinking to gain insights into peptide orientation. Information from both methods was used in modelling approaches to obtain structural models of the complexes that were identified.

Peptide binding also formed a key component of a recent study of the passage of colicin through the pore of the trimeric outer membrane porin F (OmpF) 80. Following transfer of the five‐component holo‐translocon into the mass spectrometer, which revealed that the intact complex was released from two detergent micelles, limited proteolysis was used to identify the fragment of colicin binding within the trimeric pore of OmpF. Interestingly, the identified colicin fragment was unexpectedly long, suggesting a different mechanism than simple passage through one of the trimeric OmpF pores. Synthetic peptides harbouring OmpF‐binding sites were then used to characterize binding to the pore in detail; titration of the synthetic peptides to both empty and colicin‐bound OmpF and analysis of the products by native MS revealed that two of the pores are occupied by colicin, providing insights into a novel threading mechanism in the assembly of the colicin translocon 80.

## Protein–protein interactions – establishing the correct stoichiometry

A combination of MS of intact protein complexes and quantitative proteomics is often used to define the stoichiometry of assemblies, e.g. the CRISPR interference CSM complex with eight different subunits 81. In this case, the measured mass for the intact complex was 122 kDa higher than the sum of the masses of its constituent subunits and CRISPR RNA. Whereas the CRISPR RNA is assumed to be present as a single copy, some of the protein subunits were thought to exist in multiple copies. Quantitative proteomics with a labelling approach was applied, and representative tryptic peptides from each subunit were selected for isotopic labelling at C‐terminal R/K residues. To ensure a 1 : 1 molar ratio, the peptide from the largest subunit (sso1428) was conjugated with the remaining seven peptides, resulting in seven dipeptides for synthesis. Each synthetic dipeptide was individually spiked into the CSM preparation before trypsin digestion, and the resultant peptide mixtures were analysed by LC‐MS/MS. The molar ratios of the eight CSM subunits were determined to be 4 : 3 : 1 : 1 : 1: 1 : 1 : 1, with sso1426 and sso1424 being present in four and three copies, respectively, and unit stoichiometry being seeen for the remaining subunits 81.

Similarly, determining the copy number of L7/L12 proteins in the stalk complex of the ribosome was defined either with native MS of the intact stalk complex 82 or absolute quantification approaches 84. Where a ribosome exists with stalks of different stoichiometries (heptameric and pentameric), depending on cellular conditions, the spectrum of the intact stalk complex is able to reveal the relative populations of both stoichiometries. Information about discrete populations is less obvious following decomposition of the intact ribosomes, and only average abundances have been reported from proteomics studies.

## Drug binding – defining changes by their effects on subunit interactions and dynamics

**Figure 4:**
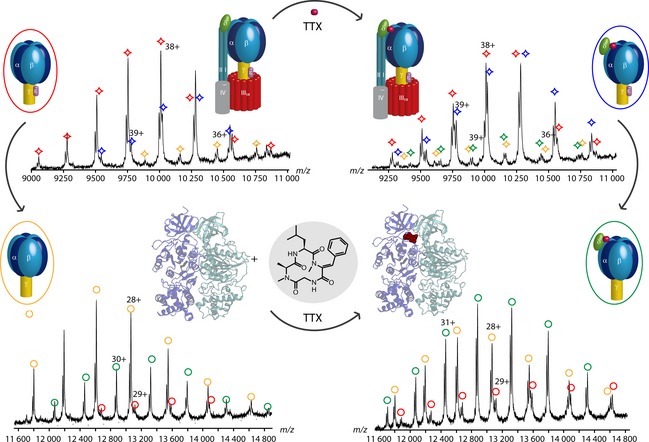
TTX binding to cATPase. The upper mass spectra were acquired before (left) and after (right) incubation with TTX. Without TTX, the dominant complex species is the F_1_–δ‐cATPase. The intact F_1_ and F_1_–δ–ε complexes are present at lower intensities. The intensity of the intact F_1_‐cATPase increased after TTX binding, leading to two main complexes, the F_1_‐cATPase and the F_1_–δ‐cATPase. Upon CID fragmentation (lower mass spectra), the ε‐subunit dissociates, mainly yielding the F_1_–δ–ε‐cATPase without TTX (left). CID fragmentation of the TTX‐bound cATPase results in an intensity change of the CID products. Loss of the ε‐subunit in the TTX‐bound complex leads primarily to formation of the F_1_–ε complex. The crystal structures of the spinach ATPase with (Protein Data Bank ID PDB 1KMH, right) and without (Protein Data Bank ID PDB 1FX0, left) TTX, as well as the structure of TTX, are shown.

The fungal cyclic tetrapeptide tentoxin (TTX) binds and specifically inhibits some chloroplast ATPases (cATPases) 85. Binding of TTX to the F_1_ head of intact F_1_‐cATPase purified from spinach leaves was studied with MS. Differences in the complexes present in solution were observed (Fig. 4). Without TTX, F_1_ loses the δ‐subunit readily to form F_1_–δ as the dominant species. After incubation with TTX, the intensity of the intact F_1_‐cATPase with the δ‐subunit increased, suggesting that the δ‐subunit binds stably to the TTX‐inhibited form of the enzyme. CID of the complexes leads primarily to loss of the ε‐subunit, yielding F_1_–δ and F_1_–δ–ε complexes (Fig. 4). Accordingly, the intensities of the CID products are different for the untreated and inhibited cATPase, implying that binding of TTX results in tighter binding of the δ‐subunit, in accord with the location of the TTX‐binding site in the X‐ray structure 86.

Studying drug binding to the ABC transporter P‐gp is complicated by the background of detergent and lipids that constantly pass through the pump 71. Having found conditions that enabled us to identify the binding of two molecules of cyclosporine A, an immunosuppressant, to P‐gp, we were able to monitor concomitant binding of lipids and drugs. Interestingly we found that drug efflux was enhanced following prior binding of cardiolipin. Moreover, using IM‐MS, we were able to distinguish changes in fluctuations of the pump, from inward‐facing to outward‐facing forms, following the binding of lipids and/or drugs. Although this approach is still in its infancy, drug binding to membrane proteins and the effects of lipids on drug binding, as well as on the dynamics of the conformational fluctuations, highlight the potential for MS to contribute to drug discovery, particularly within the challenging area of membrane protein complexes.

## Concluding remarks

It is clear from content of this review that no one MS approach is sufficient in isolation for all cases, but, rather, a combination of MS‐associated methods is needed for each application. It could be argued that changes in subunit interactions of the cATPase were revealed entirely by chemical crosslinking. However, the mechanism linking these changes to nucleotide binding was only determined by studying the extent of nucleotide binding in the intact complex. Similarly, the intact subunit stoichiometry of the CSM complex remained ambiguous following MS of the intact complex and subcomplexes generated in solution. When the results were studied in conjunction with quantitative proteomics, however, it was possible to define a unique subunit stoichiometry and to produce a model compatible with the EM density and IM CCS 81 (Fig. 1). Similarly lipid and drug binding to the ABC transporter P‐gp in isolation led to the observation of binding, but, when IM was also used, the binding could be linked to changes in the populations of inward‐facing and outward‐facing forms 71.

In this review, we have shown how MS with its diverse techniques and applications is being applied to gain insights into the structure and dynamics of protein–ligand complexes. The implementation of molecular modelling with the combination of these various approaches proved to be essential in the majority of the studies presented here. It is undoubtedly the case that continued developments in instrumentation, methodology and data analysis will enhance opportunities and widen the range of available structural information. Particularly exciting is the accessibility of highly dynamic and heterogeneous complexes that are often beyond the scope of more established structural biology approaches. Overall, we believe that, whereas the ability of MS to contribute to structural models of protein–ligand interactions are emerging, the full potential of MS in drug discovery has yet to be realised.
